# Exome-wide association study of levodopa-induced dyskinesia in Parkinson’s disease

**DOI:** 10.1038/s41598-021-99393-8

**Published:** 2021-10-01

**Authors:** Eva König, Alessandra Nicoletti, Cristian Pattaro, Grazia Annesi, Roberto Melotti, Alessandro Gialluisi, Christine Schwienbacher, Anne Picard, Hagen Blankenburg, Irene Pichler, Nicola Modugno, Marina Ciullo, Teresa Esposito, Francisco S. Domingues, Andrew A. Hicks, Mario Zappia, Peter P. Pramstaller

**Affiliations:** 1 Institute for Biomedicine, Eurac Research, Affiliated Institute of the University of Lübeck, Via Luigi Galvani 31, 39100 Bozen/Bolzano, Italy; 2 Section of Neurosciences, Department G.F. Ingrassia, University of Catania, Catania, Italy; 3 Institute for Biomedical Research and Innovation, National Research Council, Mangone (Cosenza), Italy; 4 Mediterranean Neurological Institute (MNI), IRCCS Neuromed, Pozzilli, Italy; 5 Institute of Genetics and Biophysics “Adriano Buzzati-Traverso”, National Research Council, Naples, Italy

**Keywords:** Computational biology and bioinformatics, Genetics, Molecular biology, Neuroscience, Diseases, Health care, Medical research, Molecular medicine, Neurology, Pathogenesis, Risk factors

## Abstract

Levodopa is the standard long-term dopamine replacement therapy to treat Parkinson’s disease (PD) symptoms. With time, levodopa may induce debilitating dyskinesias (LID), the treatment of which represents a large clinically unmet need. However, time-to-LID onset varies between patients, reflecting a possible genetic component. We performed an hypothesis-free whole-exome sequencing (WES)-based screening of time-to-LID onset and attempted replication of previously published candidate gene studies. A WES association analysis was carried out in 134 PD patients in a meta-analytical framework. Replication was attempted in an independent study of 97 PD patients. Variants from previously reported candidate genes (*OPRM1*, *COMT*, *BDNF*) were also specifically examined. We significantly replicated, for the first time, an association of variant rs1799971 in the *OPRM1* gene with time-to-LID onset. Furthermore, we identified two novel potentially functional variants, in the *MAD2L2* (rs2233019) and *MAP7* (rs35350783) genes*,* which were significantly associated at the discovery stage. In the replication study, the two variants showed direction-consistent effects but did not achieve the replication significance threshold. Our study provides the first WES results for time-to-LID onset, where we replicate association at *OPRM1*, and suggest new variants in *MAD2L2* and *MAP7* genes that are significant in discovery, but require larger datasets for replication. The results are being made publicly available to allow for independent external validation.

## Introduction

**Figure 1:**
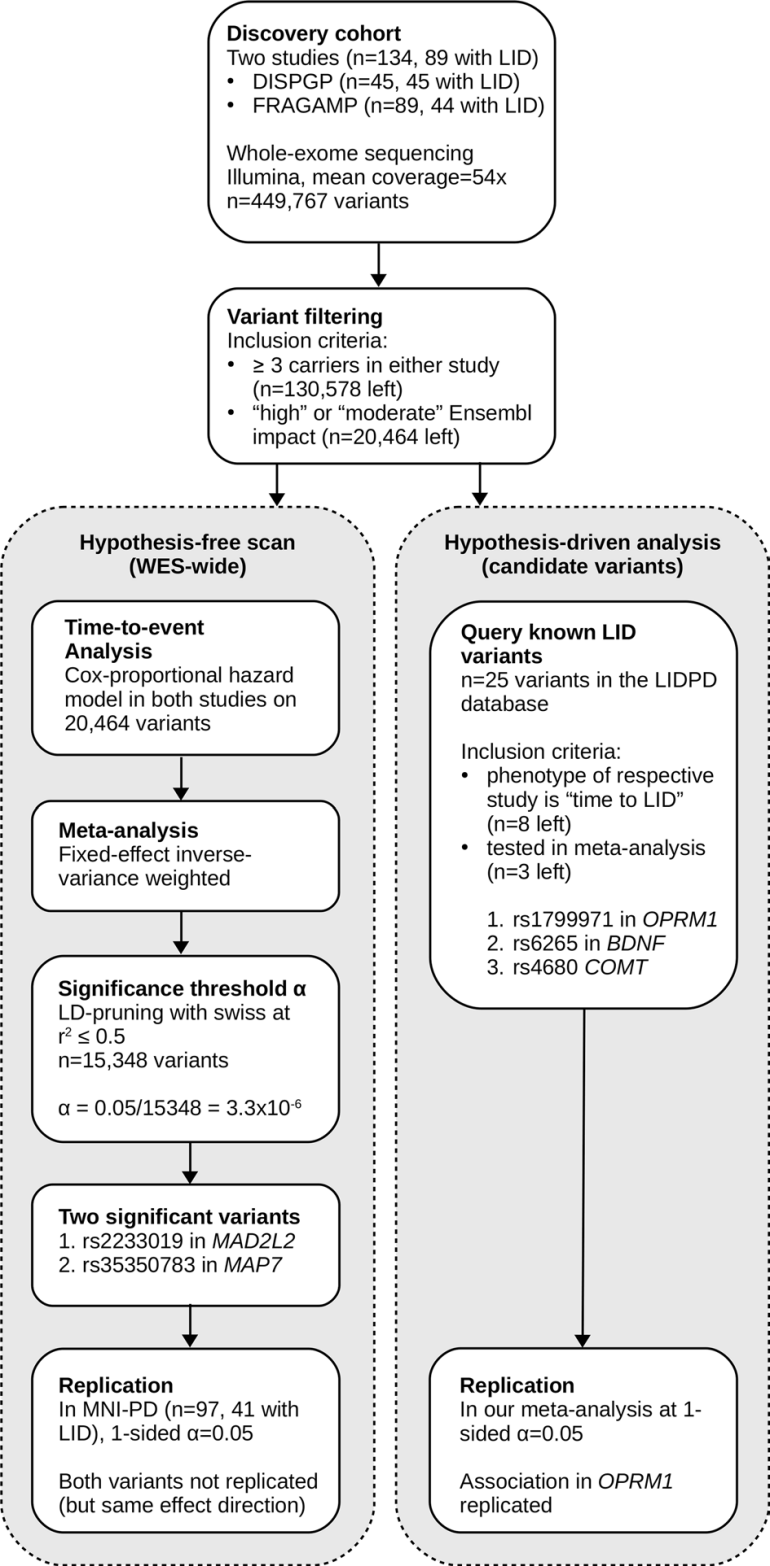
Flow chart summarizing the methodology and results of this study. Two main analyses were performed: an hypothesis-free WES-wide scan of variants associated with time to LID onset and an hypothesis-driven lookup of known candidate variants in our data.

Long recognized since the introduction of levodopa (L-dopa) for the management of Parkinson’s disease (PD), levodopa-induced dyskinesias (LID) are the most clinically challenging factors in the long-term management of PD patients. LID usually appears within 5 to 10 years after first L-dopa treatment. However, the onset of LID varies considerably among PD patients^1^. LID etiology is largely unknown and it has been attributed to various factors, including pharmacological and genetic causes. Several studies have reported associations of specific genetic variants with LID susceptibility based on candidate gene approaches with a focus on dopamine receptors and metabolism. However, candidate gene studies are prone to false positive findings^2^. To date, no hypothesis-free approach for wide disclosure of LID-associated genes has been reported. To address this gap, we conducted an exome-wide association study of time-to-LID onset on 231 PD patients (Fig. 1).

## Materials and methods

### Patients

All patients underwent a standard clinical neurological examination using the Hoehn-Yahr staging and the Unified Parkinson’s Disease Rating Scale (UPDRS-III), and were classified according to the Gelb criteria^3^. Patients with cognitive impairment, unable to sign the informed consent, or affected by atypical Parkinsonism were excluded. Presence of LID was assessed according to item 32 of the UPDRS section IV. Forty-five patients who developed LID from the DISP and GESSPARK PD cases (here referred to as DISPGP) in Bolzano, Italy, along with 89 PD patients (among which 44 developed LID) from the FRAGAMP study in Central-Southern Italy, formed the discovery cohort (n = 134)^4,5^. For replication, we used data from 97 PD patients from the MNI-PD study in Molise (among which 41 developed LID; see Table 1 and Supplementary Information for cohort details). All studies were approved by their respective ethics committees (Comitato Etico dell’Azienda Sanitaria di Provincia Autonoma di Bolzano, Comitato Etico Azienda Ospedaliera Universitaria Mater Domini di Catanzaro, Comitato Etico IRCCS INM Neuromed, Pozzilli), and were performed in accordance with current guidelines/regulations following the Helsinki Declaration and its later amendments. All participants provided written informed consent.

**Table 1:** Participant characteristics by study.

Study	Discovery			Replication	
	DISPGP	FRAGAMP		MNI-PD	
Disease status	LID	LID	Non-LID	LID	Non-LID
No. of patients passing QC	45	44	45	41	56
No. of males (%)	20 (44%)	34 (77%)	36 (80%)	23 (56%)	37 (66%)
Median follow-up time (years)^a^	5.0	4.0	5.91	4.33	2.96
Mean age at PD onset (SD)	57.8 (10.5)	54.1 (8.2)	55.0 (8.5)	51.8 (10.8)	58.9 (8.0)
Mean age at start of l-dopa treatment (SD)	60.3 (10.6)	55.1 (8.2)	56.9 (8.9)	59.8 (9.1)	61.8 (8.8)
Mean age at LID onset (SD)	65.4 (10.1)	59.6 (8.3)	NA	60.9 (9.9)	NA
Mean age at examination (SD)	68.1 (10.5)	63.2 (7.6)	62.3 (8.3)	64.5 (9.1)	66.2 (8.3)
Hoehn–Yahr scale (SD)	2.0 (0.7)	2.5 (0.7)	2.1 (0.8)	2.4 (0.6)	2.0 (0.6)

*LID* levodopa-induced dyskinesia, *SD*  standard deviation.

^a^Follow-up time is defined as the time from the beginning of levodopa treatment until LID onset or until censoring for those patients who did not develop LID. Numbers of patients reported reflect those with exome sequencing data after quality control.

### Genetic data processing

In DISPGP and FRAGAMP, WES of 144 samples was performed on an Illumina HiSeq 2500 platform. Alignment and variant calling were performed with BWA and GATK (Supplementary Information). Data quality control left 134 samples for analysis (Table 1). For each sample, an average of 50 million sequencing reads was mapped to the GRCh37 reference genome, resulting in a mean exon coverage of 54×, with ~ 79% of the target exome regions covered at ≥ 20×. In total, 449,767 variants were called by the GATK pipeline. To minimise detection of false positive associations, we limited the testing to those variants present in at least three individuals (independent of LID status) in each study (n = 130,578 variants remained). To reduce the multiple testing burden and to detect variants most likely to be functional, only variants of high and moderate impact as defined by Ensembl (see https://grch37.ensembl.org/info/genome/variation/prediction/predicted_data.html) were submitted for analysis (n = 20,464 variants in 9285 genes).

### Statistical analyses

Exome-wide scans were performed in DISPGP and FRAGAMP, separately. To assess the association with time-to-LID onset (from the beginning of l-dopa treatment), a naïve Cox proportional hazard model assuming additive genetic effects was fitted using the R package ‘survival’ version 2.43-1 (https://github.com/therneau/survival) as the main analysis. Patients who did not develop LID were censored at the time of the latest examination available for participation into each study. Neither sex, age, age at PD onset, age at LID onset, nor Hoehn-Yahr score, were independently associated with time-to-LID onset (all p-values > 0.05). A sensitivity analysis with these factors in the models did not significantly affect the reported associations of the main analysis. Results from DISPGP and FRAGAMP were pooled using a fixed-effect inverse-variance weighted meta-analysis using METAL version 2011-03-25^6^. This approach allows identification of both damaging variants associated with a shorter time-to-LID onset (positive beta) and protective variants associated with a longer time-to-LID onset (negative beta). The number of independent tests was calculated based on the linkage disequilibrium (LD) distribution across all variants, estimated with swiss version 1.0b4 (https://github.com/statgen/swiss). At the conservative LD r^2^ level of ≤ 0.5, we observed 15,348 independent variants and therefore adapted the Bonferroni-corrected significance level accordingly (α = 0.05/15,348 = 3.26 × 10^–6).^ The same models were fitted in the MNI-PD cohort, with replication tested at the one-sided α of 0.05. Finally, we assessed the association between time-to-LID onset and variants reported by previous candidate-gene studies as associated with the same phenotype. Literature screening through the LIDPD web resource^7^ identified eight variants. Of these, three were also tested in our discovery analysis: (i) rs1799971 in *OPMR1*^8^, (ii) rs6265 in *BDNF*^9^, and (iii) rs4680 in *COMT*^10,11^. Replication was tested at the one-sided α of 0.05. Figure 1 displays the methodology of this study in a flowchart.

## Results

**Figure 2:**
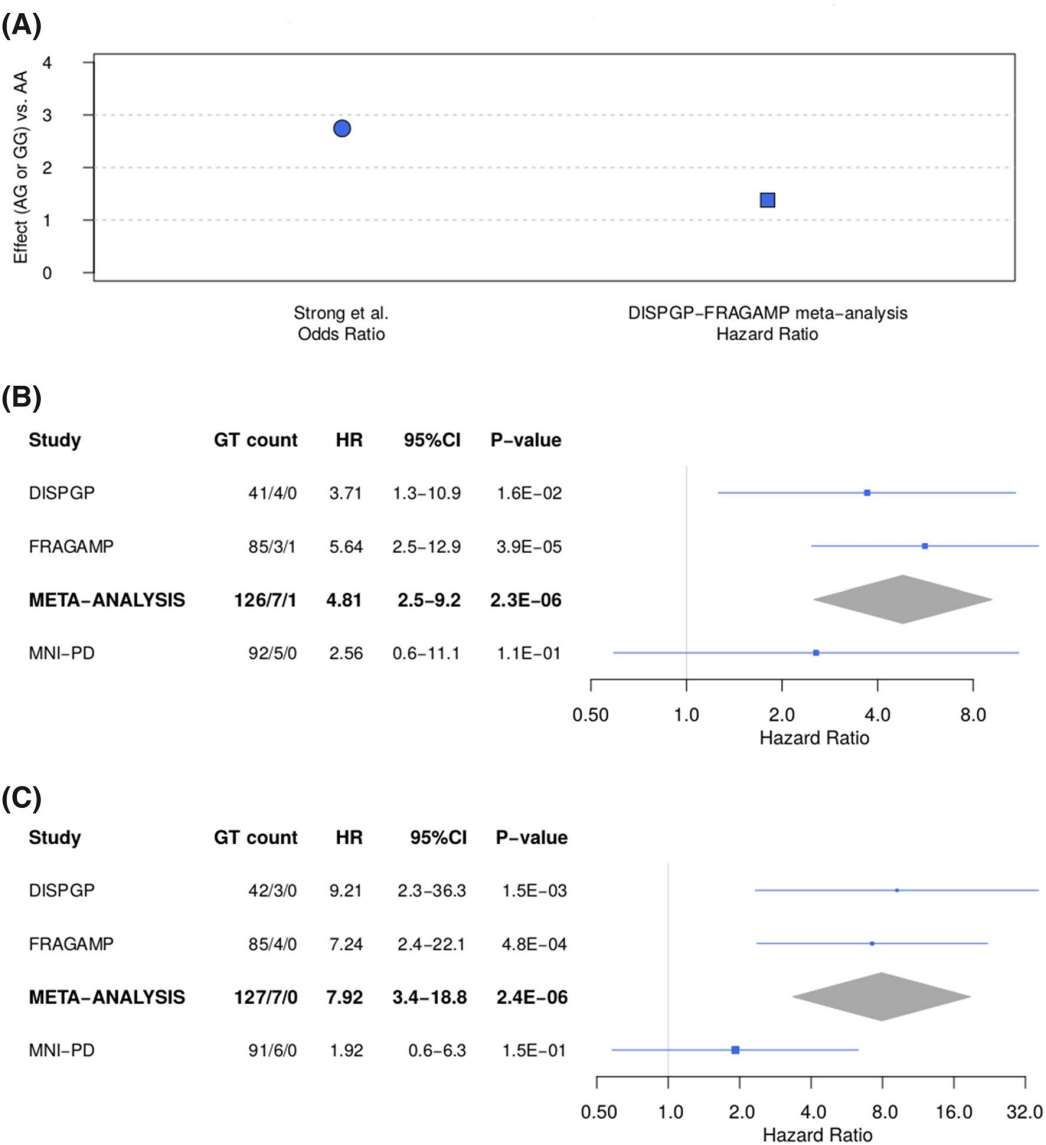
**(A)** Odds and hazard ratios of the replicated *OPRM1* variant for the effect allele G (genotypes AG or GG) versus the other allele A with genotype AA. **(B)** Forest plot of the Hazard ratio of rs2233019 in gene *MAD2L2* in the two discovery studies DISPGP and FRAGAMP, the meta-analysis, and the replication study MNI-PD. **(C)** Forest plot of the Hazard ratio of rs35350783 in gene *MAP7* in the two discovery studies DISPGP and FRAGAMP, the meta-analysis, and the replication study MNI-PD. For **(B,C)**, the point/center of the triangle corresponds to the Hazard ratio, the line/dimension of the triangle corresponds to the 95% confidence interval. *GT* genotype, *HR* hazard ratio, *CI* confidence interval.

Characteristics of study participants are described in Table 1. The whole-exome scan results, summarized in Supplementary Figure S1, supported the absence of unmodeled sample structure (estimated genomic inflation factor: 0.94; Supplementary Figure S2). When testing three variants identified in previous candidate gene studies, we replicated the association of rs1799971 in *OPRM1* (Fig. 2A, HR = 1.38, 95%CI: 1.03, + ∞, p-value = 0.038). Results of the association with all three variants are shown in Supplementary Table S2.

Two novel variants were associated with time-to-LID onset in our data (Fig. 2B,C; Supplementary Figure S1; Supplementary Table S1): the splice donor variant rs2233019 in the mitotic arrest deficient 2 like 2 (*MAD2L2*) gene (p-value = 2.3 × 10^–6^), and the missense variant rs35350783 in the microtubule associated protein 7 (*MAP7*) gene (p-value = 2.4 × 10^–6^). The minor allele (minor allele frequency, MAF = 0.034) at rs2233019 showed a hazard ratio (HR) of 4.81 (95% confidence interval, 95%CI, 2.52–9.18) with direction-consistent results in DISPGP and FRAGAMP (Fig. 2B). The minor allele (MAF = 0.026) at rs35350783 had a HR of 7.92 (95%CI, 3.35–18.77) with direction-consistent results in both DISPGP and FRAGAMP (Fig. 2C). When assessing replication in MNI-PD, direction-consistent effects for both variants were observed, even though no variant passed the replication threshold (Supplementary Table S1).

## Discussion

The current analysis presents for the first time an untargeted approach, in addition to testing previously reported associations, and adopts stringent criteria for variant selection to minimize potential false-positive signals in the genetic association with time to LID onset. We have used a discovery group comprised of 134 levodopa treated patients from two independent PD cohorts to identify exonic variants associated with time-to-LID onset. In contrast to previous candidate gene studies, which have focused largely on targets enriched for genes implicated directly in PD and parkinsonism and dopaminergic and non-dopaminergic neurotransmission, we performed an hypothesis-free exome-wide association study using high-quality filtered WES data. We only tested variants with at least three carriers in every study, which drastically reduced the number of tests. We further used stringent multiple-testing correction to minimize the risk of false positive findings. By using this approach, we replicated, to our knowledge for the first time, a previously reported association in the opioid receptor gene *OPRM1*, where in 92 adult-onset PD patients who had been taking levodopa at least 5 years and/or had developed levodopa-induced dyskinesia, carrying the G-allele of the rs1799971 A118G single nucleotide coding region polymorphism of the mu opioid receptor was independently associated with increased risk of earlier onset of dyskinesia^8^. The rs1799971(G) allele in exon 1 of *OPRM1* causes the p.Asn40Asp substitution. This influences the response to opioids such as heroin, codeine or morphine and predicts the response to naltrexone^12^. The direction of effect in our data (Supplementary Table S2) suggests that carriers of the G allele have shorter time-to-LID onset, and that therefore differential sensitivity of the mu opioid receptor to endogenous opioids could play a role in time-to-LID onset. By extension, agonists of the mu opioid receptor might be therapeutic in slowing LID onset, and can provide anti-dyskinetic benefit as has been recently discussed^13^.

Furthermore, we detected two exonic variants (rs2233019 in *MAD2L2* and rs35350783 in *MAP7*) associated with time-to-LID onset. Neither variant was formally replicated in a small independent study group. However, both variants showed consistent effect direction in the replication study, suggesting a lack of power for replication. Hence the importance of making these results available for others to attempt replication. *MAD2L2* is involved in DNA damage and repair in ageing cells^14^ with ageing being the strongest risk factor of PD. Any impairment of DNA repair systems may facilitate the onset of pathological features typical of PD including derangement of the dopaminergic system, mitochondrial dysfunction, and alpha-synuclein stress, as observed in mouse models of synucleinopathy^15^. Some of these PD-related changes, especially derangement of the dopaminergic system, may modify the time-to-LID phenotype. The *MAP7* gene codes for a microtubule-stabilizing protein expressed in many tissues, including brain. Reduced microtubule stability has been observed in several neurodegenerative diseases such as PD, Alzheimer's disease, Amyotrophic Lateral Sclerosis, and tauopathies like Progressive Supranuclear Palsy^16^, and hyperstable microtubules, as seen in Hereditary Spastic Paraplegia, also lead to neurodegeneration.

Despite plausible links to PD itself, the mechanism by which either *MAD2L2*, *MAP7*, or *OPRM1* may influence time-to-LID onset in patients is unclear, and remains speculative for now. Functional investigation in appropriate cellular models may shed insight into underlying biological mechanisms, but replicating these association findings in a larger cohort of PD patients with time-to-LID onset data would be necessary before embarking on further functional validation.

The current analysis stands out for the unbiased approach used, in addition to testing previously reported associations, and the stringent variant selection to reduce potential false-positive signals. However, our study has also several limitations, including the relatively small sample size and the heterogeneity between studies, especially in relation to patient follow-up times after starting treatment. For example, in the replication cohort MNI-PD, follow-up time for LID patients was 1.37 years longer than for the non-LID patients, raising the possibility that some non-LID patients might have developed LID during this additional time. It is also possible, that given the stringent requirement in discovery to test variants with a minimum number of three in each set of patients, we might have missed additional important variants. Data regarding l-dopa dosage was of variable quality and was not comparable between cohorts. We also did not have access to consistent data regarding LID severity, preventing us to investigate potential associations with the degree of adverse effects. Further larger patient cohorts and consistent phenotyping, along with the availability of this data for testing with other data with similar LID phenotyping, might help overcome these limitations and lead to further discovery of genetic variants associated with time-to-LID onset in PD.

To the best of our knowledge, this is the first WES analysis of time-to-LID onset. To enable independent external replication and further use of our data, we have made our results available in the LIDPD web resource^7^. It is worth noting that even in the absence of mechanistic understanding, if robustly replicated, these associated variants may help predict an individual’s risk of developing LID, and may indicate those at risk of developing LID more quickly. Such information could allow tailored therapies to lessen the impact of LID. At best, discovery of these genes indicate possible novel drug targets or pathways to delay, counteract or even negate LID development. For the latter, our results support further investigation of mu opioid receptor drugs as modifiers of LID, especially in patients carrying the G allele of rs1799971.

## Supplementary Information

Supplementary Information.
