# Supplementary material for: Exome-wide association study of levodopa-induced dyskinesia in Parkinson’s disease
Source: Sci Rep. 2021 Oct 1;11:19582. doi: 10.1038/s41598-021-99393-8 (PMC8486836; doi:10.1038/s41598-021-99393-8)
Supplement: Supplementary file 1 — Supplementary Information. [file 41598_2021_99393_MOESM1_ESM.docx]

**Supplementary Information**

**Paper Title**

**Exome-wide association study of levodopa-induced dyskinesia in Parkinson's disease**

Eva König PhD^1^, Alessandra Nicoletti MSc^2^, Cristian Pattaro PhD^1^, Grazia Annesi PhD^3^, Roberto Melotti PhD^1^, Alessandro Gialluisi PhD^4^, Christine Schwienbacher PhD^1^, Anne Picard BSc^1^, Hagen Blankenburg PhD^1^, Irene Pichler PhD^1^, Nicola Modugno MD^4^, Marina Ciullo PhD^4,5^, Teresa Esposito PhD^4,5^, Francisco S. Domingues PhD^1^, Andrew A. Hicks PhD^1*§^, Mario Zappia MD^2*^, Peter P. Pramstaller MD^1*^

^1^ Institute for Biomedicine, Eurac Research, Affiliated Institute of the University of Lübeck, Bolzano, Italy

^2^ Department G.F. Ingrassia, Section of Neruosciences, University of Catania, Catania, Italy

^3^ Institute for Biomedical Research and Innovation, National Research Council , Mangone CS, Italy.

^4^ Mediterranean Neurological Institute (MNI), IRCCS Neuromed, Pozzilli, Italy

^5^ Institute of Genetics and Biophysics "Adriano Buzzati-Traverso", National Research Council, Naples, Italy

^*^ Equal contributions

^§^ To whom correspondence should be addressed at:

Institute for Biomedicine

Via Luigi Galvani 31

39100 Bozen/Bolzano

Italy

andrew.hicks@eurac.edu

**Ethics approval**

The local ethics committee (Comitato etico del comprensorio sanitario di Bolzano; reference numbers: 2008-D2-001090, 62/2012, 58/2013, 59/2013) approved the studies in Bolzano, and local ethics committees approved the other studies. All participants provided written informed consent.

**Genetic Data processing**

DISPGP (n=48) and FRAGAMP (n=96) samples were exome enriched following the recommended procedures of either the Nextera Rapid Capture kit or TruSeq v1.2 protocols (Illumina). The resulting exome libraries were sequenced on an Illumina HiSeq 2500 in paired-end mode. Adapter sequences were trimmed off using SeqPrep (https://github.com/jstjohn/SeqPrep) and read quality was controlled with FastQC (http://www.bioinformatics.babraham.ac.uk/projects/fastqc/). Reads were aligned to reference genome GRCh37 with BWA version 0.7.15 ^1^. Duplicate reads were marked with picardtools version 2.8.1 (https://broadinstitute.github.io/picard/). Indels were realigned and base quality scores recalibrated using GATK 3.7 ^2^, following the GATK best practice guidelines ^3^. Quality of bam files was evaluated with QualiMap version 2.2.1 ^3^, sample contamination estimated with verifyBamId ^4^ and sex validated by inspection of the X chromosome coverage. Intermediate per sample gvcf files were generated with the GATK HaplotypeCaller, followed by joint genotyping on all individuals with GATK GenotypeGVCFs. Variants were annotated with Ensembl gene and variant consequence data using the Dintor gcoords2cons tool ^5^. Following quality control, data from 45 DISPGP samples and 89 FRAGAMP samples was available for subsequent discovery analysis.

Variants were classified as non-synonymous, if their consequence was either of “high” (transcript ablation, splice acceptor variant, splice donor variant, stop gained, frameshift variant, stop lost, start lost, transcript amplification) or “moderate” impact (in-frame insertion, in-frame deletion, missense variant, protein altering variant), according to release 75 of Ensembl, considering the most severely affected transcript per gene. Furthermore, variants were required to be present in homozygous or heterozygous state in at least three individuals in both sub-studies, which was the case for 20,464 variants in 9,285 genes. These variants were tested for association with LID.

**Replication cohort: the MNI-PD study**

The MNI-PD cohort consists of 114 (42 familial and 72 sporadic) PD cases recruited at IRCCS Neuromed, Pozzilli (IS), Italy between June 2015 and June 2016. These samples underwent Whole Exome Sequencing (WES) at Helmotz Zentrum, Munich, Germany. Genomic DNA was isolated from peripheral blood lymphocytes by Blood and Cell Culture DNA Midi Kit (QIAGEN, Hilden, Germany). Exonic regions were enriched using the SureSelect All Exome kit v6 (Agilent® Technologies, Santa Clara, CA, USA) based on DNA fragmentation and capture. Exomes were barcoded and sequenced using the Illumina® HiSeq2000 platform (Illumina, San Diego, CA, USA).

The alignments of the 100-bp paired-end reads to the human reference genome was performed through BWA MEM v0.7.542. ^1^ After removal of duplicate reads through Picard, single nucleotide variants (SNVs) and insertions/deletions (indels) were called, using HaplotypeCaller and GenotypeGVCFs in GATK v3.5-0-g36282e4. ^2^ Average exome coverage was 143x and at least 20x for 98.8% of the target. Of these samples, we retained for analysis only participants with complete information on LID status and follow-up time (N=97, 41 LID) and extracted genotype data of the two SNPs which were assessed for replication, namely rs2233019 and rs35350783 (call rate 100% for both SNPs).

**Supplementary Figure S1.**

**
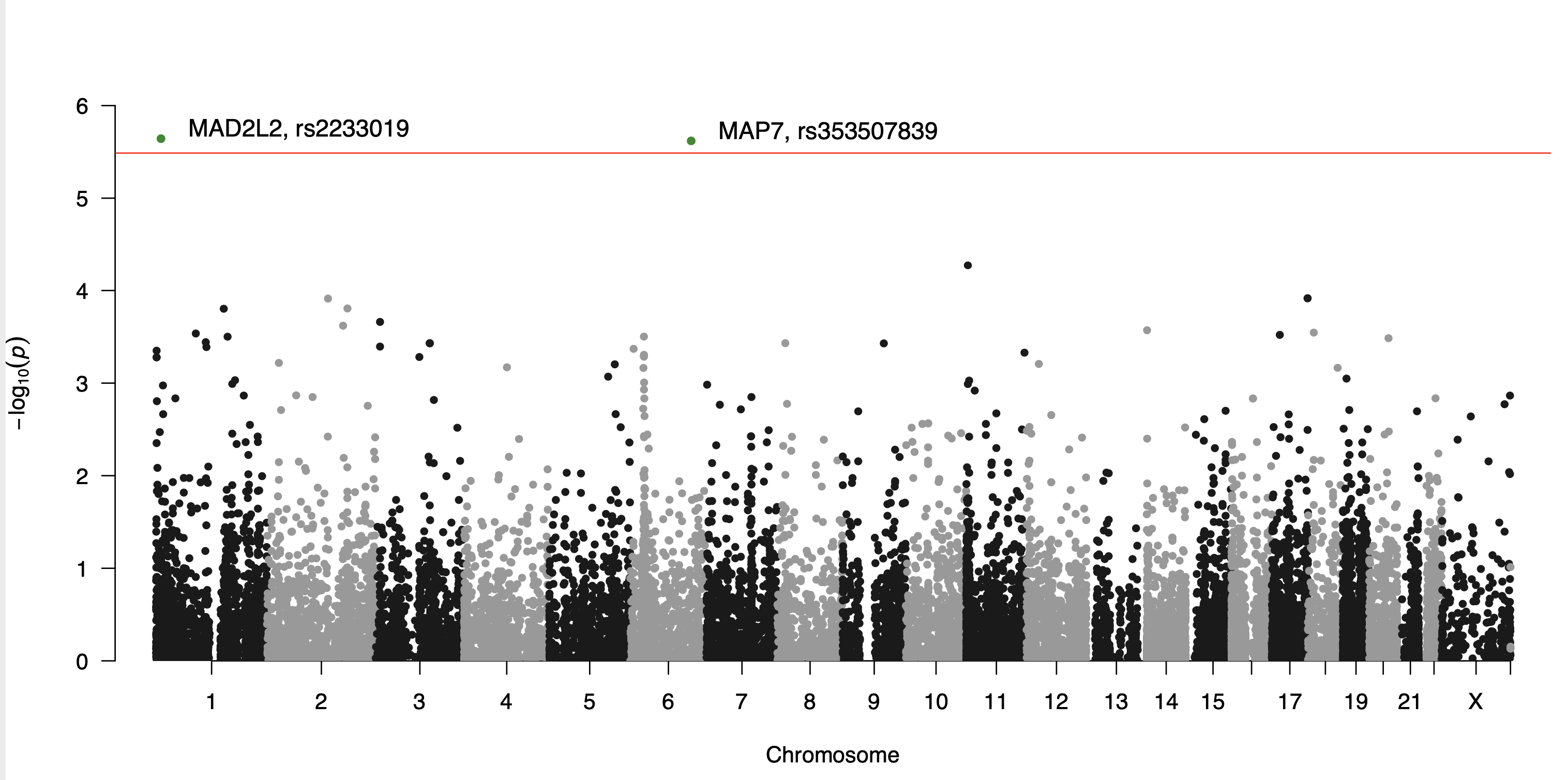
**

**Supplementary Figure S1.** Manhattan plot of the discovery exome-wide meta-analysis results. Displayed are the –log_10_ (p-values) for association between time-to-LID onset and each genetic variant. The horizontal red line indicates the significance threshold at 3.26x10^-06^ (Methods). Green dots highlight the exome-wide significant variants. The plot was generated with the ‘qqman’ R-package version 0.1.4 (https://cran.r-project.org/web/packages/qqman/index.html).

**Supplementary Figure S2.**

**
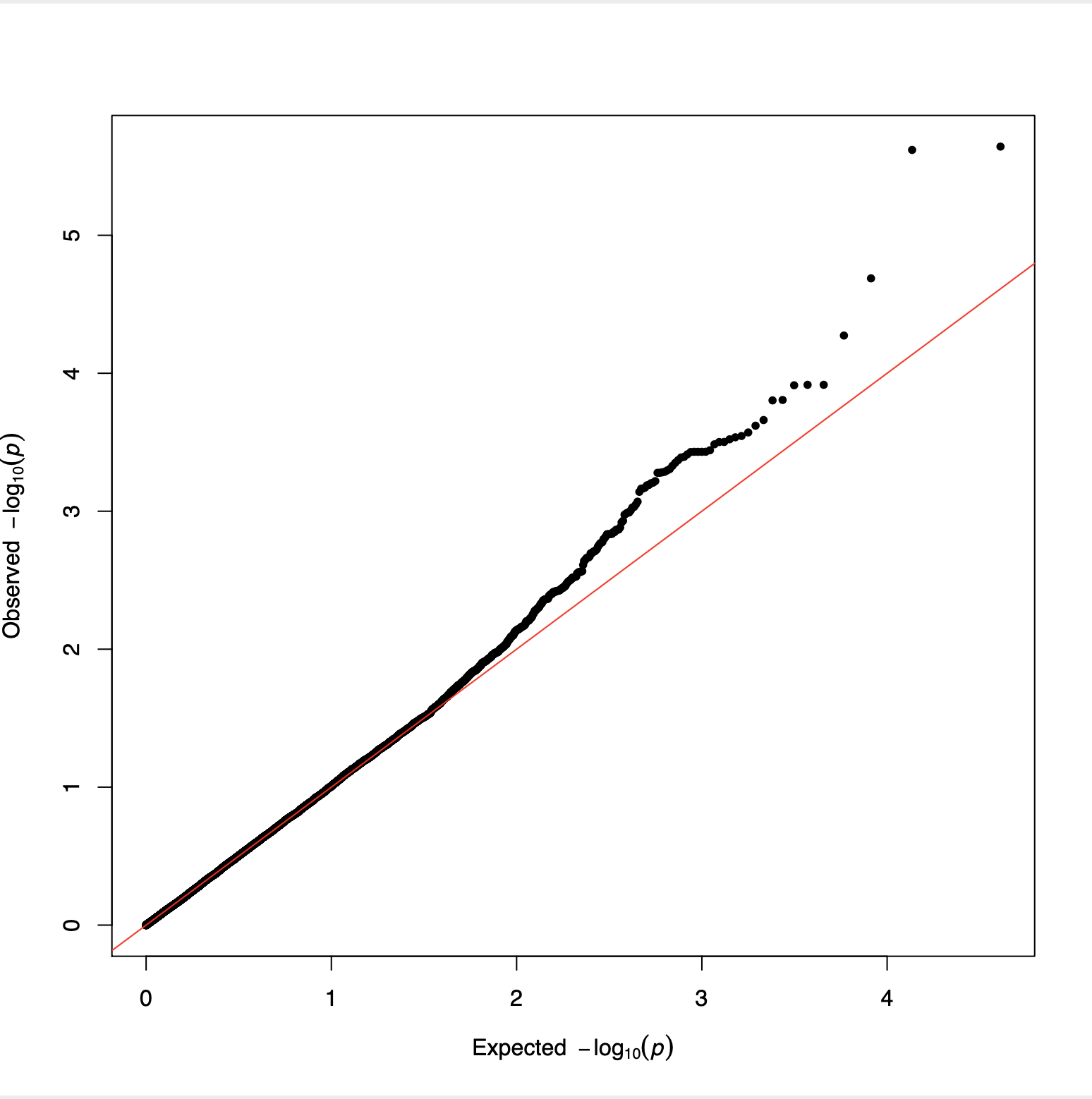
**

**Supplementary Figure S2.** QQ-plot of expected versus observed –log_10_ (p-values), generated with the ‘qqman’ R-package version 0.1.4 (https://cran.r-project.org/web/packages/qqman/index.html). Genomic inflation factor λ = 0.94.

| **dbSNP ID** | **Genomic Coordinates^a^** | **Effect Allele** | **Other Allele** | **Variant Consequence** | **Gene** | **Analysis** | **Beta** | **SE** | **HR** | **95%CI** | **P-value^b^** | **I^2^** | **Genotype Count (0/1/2)^c^** | **Effect allele frequency** |
| --- | --- | --- | --- | --- | --- | --- | --- | --- | --- | --- | --- | --- | --- | --- |
| rs2233019 | 1:11736864 | C | A | splice donor | *MAD2L2* | Discovery DISPGP | 1.31 | 0.55 | 3.71 | 1.26-10.89 | 6.2E-03 |  | 41/4/0 | 0.044 |
|  |  |  |  |  |  | Discovery FRAGAMP | 1.73 | 0.42 | 5.64 | 2.48-12.85 | 3.9E-05 |  | 85/3/1 | 0.028 |
|  |  |  |  |  |  | Discovery meta-analysis | 1.57 | 0.33 | 4.81 | 2.52-9.18 | 2.3E-06 | 0.0 | 126/7/1 | 0.034 |
|  |  |  |  |  |  | Validation MNI-PD | 0.94 | 0.75 | 2.56 | 0.59-11.13 | 1.1E-01 |  | 92/5/0 | 0.026 |
|  |  |  |  |  |  | Meta-analysis DISPGP- FRAGAMP-MNI-PD | 1.47 | 0.30 | 4.35 | 2.42-7.83 | 1.4E-05 | 0.0 | 218/12/1 | 0.030 |
| rs35350783 | 6:136687065 | T | C | missense | *MAP7* | Discovery DISPGP | 2.22 | 0.70 | 9.21 | 2.33-36.31 | 1.5E-03 |  | 42/3/0 | 0.033 |
|  |  |  |  |  |  | Discovery FRAGAMP | 1.98 | 0.57 | 7.24 | 2.37-22.14 | 4.8E-04 |  | 85/4/0 | 0.022 |
|  |  |  |  |  |  | Discovery meta-analysis | 2.07 | 0.44 | 7.92 | 3.35-18.77 | 2.4E-06 | 0.0 | 127/7/0 | 0.026 |
|  |  |  |  |  |  | Validation MNI-PD | 0.65 | 0.61 | 1.92 | 0.58-6.33 | 1.5E-01 |  | 91/6/0 | 0.031 |
|  |  |  |  |  |  | Meta-analysis DISPGP- FRAGAMP-MNI-PD | 1.59 | 0.36 | 4.90 | 2.42-9.93 | 8.6E-06 | 45.2 | 218/13/0 | 0.028 |

**Supplementary Table S1.** Details of the two variants achieving significance in the discovery meta-analysis.

***Abbreviations***: **Beta** = effect (log-hazard ratio) on the time to LID onset referred to a one-copy increase of the effect allele; **SE** = standard error.

^a^ Genomic coordinates given in GRCh37.

^b^ Two-sided p-value for all analysis, except for the “Validation MNI-PD”, where the one-sided p-value is given.

^c^ Genotype count, where 0 represents genotype other/other, 1 represents genotype other/effect, and 2 represents genotype effect/effect.

**Supplementary Table S2.** Replication of known LID associated variants obtained from the LIDPD Webserver ^6^, ordered by chromosomal position.

|  |  |  |  |  | **Reference study** | | | | | | **DISPGP-FRAGAMP meta-analysis** | | | | | |
| --- | --- | --- | --- | --- | --- | --- | --- | --- | --- | --- | --- | --- | --- | --- | --- | --- |
| **dbSNP ID** | **Genomic Coordinates^a^** | **OA** | **EA** | **Gene** | **Phenotype** | **Genotype** | **Type of Relative Risk estimator** | **Estimate** | **95%CI** | **Ref** | **Beta** | **SE** | **HR** | **95%CI^§^** | **One-sided p-value** | **Direction of effect (DISPGP, FRAGAMP)** |
| rs1799971 | 6:154360797 | A | G | *OPRM1* | early vs. late LID onset | AA vs. (AG or GG) | OR | 2.78 | 1.03-7.51* | ^7^ | 0.32 | 0.18 | 1.38 | 1.03 - inf | 0.0384 | ++ |
| rs6265 | 11:27679916 | C | T | *BDNF* | time to LID onset | CC vs. (CT or TT) | HR | 2.12 | 1.36-3.38 | ^8^ | 0.11 | 0.20 | 1.12 | 0.81 - inf | 0.2969 | +- |
| rs4680 | 22:19951271 | G | A | *COMT* | time to LID onset | GG vs. (GA or AA) | HR | 2.52 | 1.29-4.95 | ^9^ | 0.26 | 0.16 | 1.30 | 1.00 - inf | 0.0515 | -+ |

**Supplementary Table S2.** Replication of known LID associated variants obtained from the LIDPD Webserver ^6^, ordered by chromosomal position.

**References**

1 Li, H. & Durbin, R. Fast and accurate long-read alignment with Burrows-Wheeler transform. Bioinformatics 26, 589-595, doi:10.1093/bioinformatics/btp698 (2010).

2 DePristo, M. A. et al. A framework for variation discovery and genotyping using next-generation DNA sequencing data. Nat Genet 43, 491-498, doi:10.1038/ng.806 (2011).

3 Garcia-Alcalde, F. et al. Qualimap: evaluating next-generation sequencing alignment data. Bioinformatics 28, 2678-2679, doi:10.1093/bioinformatics/bts503 (2012).

4 Jun, G. et al. Detecting and estimating contamination of human DNA samples in sequencing and array-based genotype data. Am J Hum Genet 91, 839-848, doi:10.1016/j.ajhg.2012.09.004 (2012).

5 Weichenberger, C. X. et al. Dintor: functional annotation of genomic and proteomic data. BMC Genomics 16, 1081, doi:10.1186/s12864-015-2279-5 (2015).

6 Blankenburg, H. et al. A Web Resource for Levodopa-Induced Dyskinesia Genetics in Parkinson's Disease. Neuroinformatics 15, 297-300, doi:10.1007/s12021-017-9327-z (2017).

7 Strong, J. A. et al. Genotype and smoking history affect risk of levodopa-induced dyskinesias in Parkinson's disease. Mov Disord 21, 654-659, doi:10.1002/mds.20785 (2006).

8 Foltynie, T. et al. BDNF val66met influences time to onset of levodopa induced dyskinesia in Parkinson's disease. J Neurol Neurosurg Psychiatry 80, 141-144, doi:10.1136/jnnp.2008.154294 (2009).

9 de Lau, L. M., Verbaan, D., Marinus, J., Heutink, P. & van Hilten, J. J. Catechol-O-methyltransferase Val158Met and the risk of dyskinesias in Parkinson's disease. Mov Disord 27, 132-135, doi:10.1002/mds.23805 (2012).
